# Measuring visual ability in linguistically diverse populations

**DOI:** 10.3758/s13428-024-02579-x

**Published:** 2024-12-30

**Authors:** Madison A. Hooper, Andrew Tomarken, Isabel Gauthier

**Affiliations:** Department of Psychology, Vanderbilt University, Nashville, TN USA

**Keywords:** Object recognition, Individual differences, Measurement, High-level vision, Spanish, English

## Abstract

Measurement of object recognition (OR) ability could predict learning and success in real-world settings, and there is hope that it may reduce bias often observed in cognitive tests. Although the measurement of visual OR is not expected to be influenced by the language of participants or the language of instructions, these assumptions remain largely untested. Here, we address the challenges of measuring OR abilities across linguistically diverse populations. In Study 1, we find that English–Spanish bilinguals, when randomly assigned to the English or Spanish version of the novel object memory test (NOMT), exhibit a highly similar overall performance. Study 2 extends this by assessing psychometric equivalence using an approach grounded in item response theory (IRT). We examined whether groups fluent in English or Spanish differed in (a) latent OR ability as assessed by a three-parameter logistic IRT model, and (2) the mapping of observed item responses on the latent OR construct, as assessed by differential item functioning (DIF) analyses. Spanish speakers performed better than English speakers, a difference we suggest is due to motivational differences between groups of vastly different size on the Prolific platform. That we found no substantial DIF between the groups tested in English or Spanish on the NOMT indicates measurement invariance. The feasibility of increasing diversity by combining groups tested in different languages remains unexplored. Adopting this approach could enable visual scientists to enhance diversity, equity, and inclusion in their research, and potentially in the broader application of their work in society.

## Introduction

Compared to the long history of studying individual differences in psychology (Cronbach & Meehl, 1955; Spearman, 1904), the measurement of high-level visual abilities in a normal adult population is a more recent endeavor (Dennett et al., 2012; Gauthier et al., 2022; McGugin et al., 2012; Richler et al., 2019; Wilmer, 2008). One important high-level visual ability is object recognition. This is the ability to discriminate between visually similar objects, which contributes to performance on a variety of tasks including matching, memory judgments or even judgments about summary statistics for groups of objects (Richler et al., 2017, 2019; Sunday et al., 2021). The novel object memory tests (NOMTs, Richler et al., 2017) are simple tests that avoid the influence of category-specific experiences on visual tasks (e.g., birding classes or attending car shows). According to one estimate, almost none of the variance shared between different NOMTs is explained by IQ (Richler et al., 2017). NOMTs have been used as indicators of a domain-general ability that contributes unique variance (beyond IQ) to the prediction of complex visual tasks like reading musical notation (Chang & Gauthier, 2021) or learning to make a variety of medical decisions that rely on visual information (Holmes et al., 2020; Smithson et al., 2022; Sunday et al., 2018a, 2018b).

If object recognition is central to many activities valued in society, including those that keep us safe and healthy, or allow us to learn new skills, we may benefit from its use in aptitude testing. Abilities that dissociate from intelligence like object recognition can increase precision of prediction and reduce some of the social inequities in current aptitude testing (Carroll et al., 2019; Skiba et al., 2002). Adverse impact through the inequities that can arise in cognitive testing influences professional opportunities and economic outcomes (Burgoyne et al., 2021). Object recognition may predict learning and success in some occupations, reducing the bias that is often observed in cognitive tests (Bobko & Roth, 2013). There is no a priori reason to expect that object recognition measures should be biased according to gender or ethnicity, and these tests cannot be easily adapted in different languages, given they include minimal verbal instructions. With the exception of data on age and gender (Richler et al., 2017), there is little known about potential sources of bias, and the role of language in visual tests has not been studied before.

In 2021, Hispanics accounted for nearly one in five people in the United States (Krogstad et al., 2023). The proportion of Latinos in the United States who speak English proficiently is growing, but a majority (68% of all Latinos and 93% of foreign-born Latinos in 2021) report speaking Spanish at home. Our goal is to validate the use of NOMTs regardless of the language of the instructions, English versus Spanish, and in particular, evaluate whether the same OR ability can be measured in English-speaking and Spanish-speaking populations.

In Study 1, we tested the assumption that language of instructions does not matter to this task by measuring whether similar overall performance can be achieved on the NOMTs when English–Spanish bilinguals are randomly assigned to one or the other version. Study 1 represents a sanity check because we do not expect the specific instructions in a visual task like the NOMT to be theoretically important. We therefore expect the language of instructions should have no effect. We use Bayesian tests appropriate to evaluate the support for this prediction. One reason it is important to verify that the language of instructions causes no difference is because visual studies generally never combine data collected in different languages. Psychology lore contains many examples of small manipulations with important effects, and scientists may be reluctant to simply assume that it does not matter. A second reason is that in Study 2, we build on these results and compare two groups that differ in *both* their language fluency and the language of testing. Going into this study with confirmation that our Spanish and English instructions are equivalent, when the participants are randomly assigned, will facilitate interpretation.

Then, in Study 2, we dig deeper into a subset of the NOMTs tested in Study 1 (two of the three tasks, the most reliable) to address a second assumption which is that the participants who speak Spanish or English should not differ in their NOMT performance. We first discuss a mean difference in performance between groups, a possible explanation for this difference, and the implications for our analyses. We then move to using a method that should allow us to better interpret this difference—is the test measuring the same ability in the two groups of participants? For this purpose, we used item response theory (IRT).

Ultimately, the goal of the entire project is to we ask whether groups of individuals tested in English versus Spanish NOMTs could be justifiably pooled together to study visual abilities. This would provide a proof of concept that a visual object recognition task administered in different languages measures the same ability, and it may encourage greater diversity in samples tested in visual studies.

## Study 1

### Participants

Spanish–English bilingual participants were recruited on Amazon Mechanical Turk and were restricted to those with an approval rate of 98% or more with 5000 or more tasks approved. All participants provided informed consent, which was written in English and in Spanish. The data were collected in June 2021. Participants were paid $3.50 for about 25 min of time spent on the tasks ($8.4/hr). We included five attention checks in the study (where the two distractor objects were from very different categories in different colors, whereas all relevant objects were yellow). We excluded participants if they did not correctly answer at least four attention checks. In total, including all participants who completed the study (*N* = 145) and passed the attention checks (*N* = 119), we have 63 in the English instructions and 56 in the Spanish instructions condition.

The demographics in each instruction group were as follows: Spanish instructions: mean age = 30.75, *SD* = 8.79, 4 not reporting age; 30 women, 33 men; English instructions: mean age = 30.58, *SD* = 8.89, 3 not reporting age; 27 women, 33 men, 1 transgender. Most participants were from three countries (English instructions group: 28 USA, 19 India, 10 Brazil, 2 other, 4 not reporting; Spanish instructions group: 23 USA, 18 India, 8 Brazil, 4 other, 3 not reporting). We also asked participants in each instruction group to report their fluency with written English and Spanish from 0 (no knowledge) to 4 (native reader). Fluency in the English instructions group was as follows: English fluency = 3.19, *SD* = 1.12; Spanish fluency = 2.92, *SD* = 1.12. Fluency in the Spanish instructions group was as follows: English fluency = 2.93, *SD* = 1.25; Spanish fluency = 2.64, *SD* = 1.40. Bayesian independent-samples *t*-tests (see *Analyses* for details) found little evidence for a difference between the groups in Spanish fluency (*BF*_10_ = 0.362) or English fluency (*BF*_10_ = 0.376). There was inconclusive evidence for a difference in the fluency of the relevant language tested for each group (English for those receiving English instructions and Spanish for those receiving Spanish instructions, *BF*_10_ = 2.28).

### Measures

**Fig. 1:**
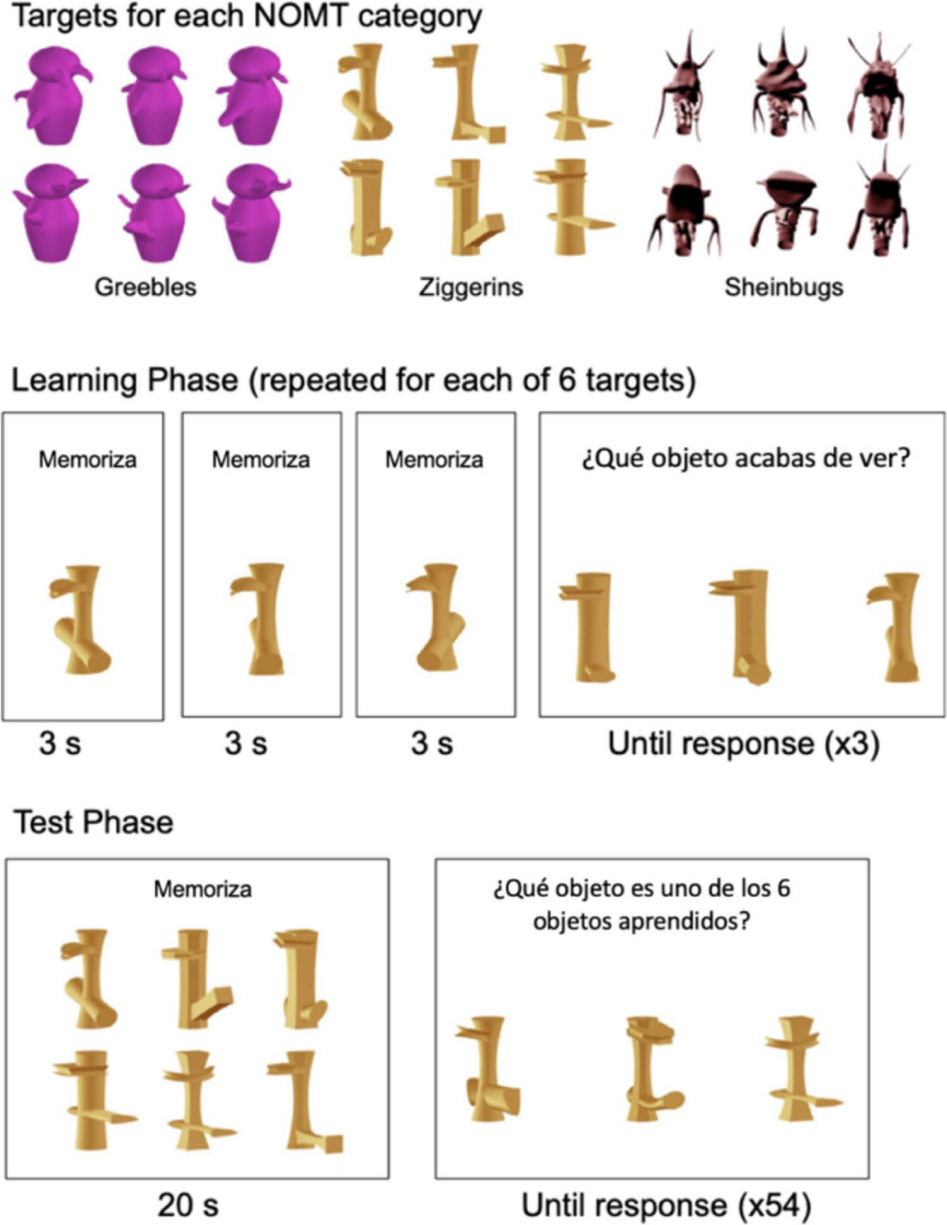
Objects for the three NOMTs used in Experiment 1 structure of trials for the learning and test phases, with Spanish instructions

We adapted the three novel object memory tests (NOMTs) from Richler et al. (2017), with novel categories called Greebles, Ziggerins, and Sheinbugs (see Fig. 1). We created a new version with Spanish instructions. The trial order is slightly different from that in the Richler et al. (2017) study but is identical in our Spanish and English versions.

Participants completed three NOMTs (with Ziggerins, then Greebles, then Sheinbugs), each including 72 trials. The first NOMT began with a practice section using artificial Lego® objects. The following instructions were shown: “Memorize the object in the next 3 images. A test will follow”/“Memoriza el objeto en las próximas 3 imágenes. Una prueba seguirá.” Then, three different nonsense Lego® objects were shown for 3 s each, with the instruction “memorize”/ “memoriza.” Three trials followed, each showing a choice of three new objects with the following instructions: “Which object did you just view? There is only 1 correct answer. Click on the correct object”/ “Qué objeto viste? Solo Hay 1 respuesta correcta. Apriete el objeto correcto.”

After the three practice trials (on which participants received correct/incorrect feedback), they saw the instructions: “Let’s do the actual test. You will learn to recognize 6 objects”/”Hagamos la prueba real. Aprenderá a reconocer 6 objectos.” The learning phase (trials 1–18) used the same format and instructions to teach the six targets for the test. On each trial, a single target object of the category was shown from three different angles with 3 s per angle (Fig. 1B). This was followed by three tests showing three similar but different objects, and participants had to select which they had previously studied. This was repeated for each of the six target objects. Next, participants had 20 s to study the six target objects simultaneously (with instructions “memorize”/”memoriza”). In the following 54 test phase trials (block 2: trials 19–48; block 3: trials 48–72), participants had to select which of the three objects shown was one of the six target objects. Targets and distractors were shown from the same angle within each test trial. Participants had another 20-s study period between blocks 2 and 3.

The learning phase for the next NOMT (with Greebles, then with Sheinbugs) began immediately after the last trial of the previous test. Trials were not timed, and each item was scored as 0 (incorrect) or 1 (correct). Chance performance on this task was equal to 0.33.

### Analyses

We used JASP (JASP Team, 2023) to conduct independent-samples Bayesian *t*-tests comparing for each of the three tasks, testing the hypothesis that the two groups did not differ against the alternative model that they do differ. Bayesian hypothesis testing allows the comparison between competing models to evaluate which one is best supported by the data. This approach is particularly well suited to this study because it allows us to quantify support not only for a difference between groups, but also for the null hypothesis, which we expected. The *BF*_10_ represents the likelihood for a difference (*H*_1_) over no difference (*H*_0_), while *BF*_01_ represents the likelihood of *H*_0_ over *H*_1_. *BF*_+0_ denotes the evidence for a positive correlation relative to a correlation ≤ 0. Bayes factors can be interpreted without any arbitrary cutoff, but we follow conventions set out by Jeffreys (1961) to describe the magnitude of evidence: anecdotal (*BF* = 1–3), substantial (*BF* = 3–10), strong (*BF* = 10–30), very strong (*BF* = 30–100), and decisive (*BF* > 100). Bayesian analyses require the specification of priors that represent the initial belief about parameters before considering the data. For *t*-tests, we adopted the non-informative default Cauchy prior centered at 0 with an interquartile range of 0.707. For correlations, we used a beta (1,1) prior stretched to encompass the range between − 1 and + 1). This choice of priors is common, as it provides a balance between being weakly informative and allowing the data to predominantly influence the posterior estimates.

### Results

The datasets (Gauthier et al., 2023) generated during and analyzed during the current study are available in the figshare repository: 10.6084/m9.figshare.24395098.v1. Mean performance, reliability, skewness, and kurtosis are presented in Table 1 for each task as a function of group. As expected from prior work (Richler et al., 2017, 2019), reliability was near or above 0.80 in all cases.

**Table 1:** Descriptive statistics for individual tasks as a function of instruction group

	*M*	*SD*	Reliability	Skewness	Kurtosis
Greebles—Spanish Instructions	.593	.131	.847/.862	0.168	− 0.539
Greebles—English Instructions	.588	.126	.836/.852	0.189	0.344
Ziggerins—Spanish Instructions	.686	.170	.919/.926	0.100	− 0.924
Ziggerins—English Instructions	.676	.170	.919/.925	− 0.256	− 0.743
Sheinbugs—Spanish Instructions	.560	.115	.796/.819	1.075	2.403
Sheinbugs—English Instructions	.561	.104	.753/.780	0.297	0.373

Reliability is Cronbach alpha/Guttman lambda2

In all three cases, the *BF*_01_ was above 3, offering substantial evidence for no difference as a function of instructions (Greebles *BF*_01_ = 5.003; Ziggerins *BF*_01_ = 5.113; Sheinbugs *BF*_01_ = 4.892). Similar results were obtained for a range of Cauchy prior widths, from 0.5 to 1.5. Note that we do not compare performance between tests, as there was no formal effort to equate their difficulty. In Table 2, we present the correlations (with 95% credible intervals) for each pair of tests in the two groups. These correlations are numerically larger than those reported for English-only versions by Richler et al. (2017) in samples of ~ 325 participants (*r*-values for pairwise were between 0.43 and 0.50). In that study, English-reading participants volunteered on an online platform and participated without any compensation (they may therefore have been particularly motivated). Consistent with this possibility, mean performance on the tests in Richler et al. (2017) was on average 14% higher than in this study. As expected, Bayesian tests provided very strong evidence for positive correlations (all *BF*_+0_ > 1.10E + 06, and these results did not change qualitatively over a very large range of beta priors). More interesting is the large overlap between 95% credible intervals, suggesting no evidence that the instruction language influences the correlations between the tests.

**Table 2:** Correlations between tests, for the English (top) and Spanish (bottom) instruction groups

	Greebles	Sheinbugs
Sheinbugs	.704 (.540; .804)	
	.662 (.469; .780)	
Ziggerins	.772 (.636; .851)	.647 (.464; .765)
	.741 (.579; .834)	.743 (.582; .836)

### Discussion

In Experiment 1 we compared the performance of Spanish–English bilingual individuals on three tests of OR ability, which had the same format but used different categories of novel objects. Our goal was to demonstrate that the Spanish and English instructions were comparable. We found evidence supporting the absence of difference in mean performance as a function of instruction language. The robust correlations across tests, which are thought to largely tap into the same visual ability (Richler et al., 2019; Sunday et al., 2021), also do not seem to be impacted by language of instructions.

## Study 2

In Experiment 2, we extended our previous work by incorporating several methodological advancements. We recruited a larger sample and used a between-groups design, with participants categorized based on their proficiency in either English or Spanish. Additionally, we employed item response theory (IRT) for a more nuanced psychometric evaluation (e.g., de Alaya, 2009; Embretson & Reise, 2000). Unlike Experiment 1, where a single group of participants received instructions in either English or Spanish, Experiment 2 had distinct groups and languages for instructions. This created two potential threats to measurement invariance, which is an important prerequisite for meaningful group comparisons (Stark et al., 2006). In the IRT literature, a lack of measurement invariance is referred to as differential item functioning (DIF). The aim of Experiment 1 had been to establish that language of instruction (English vs. Spanish), on average, did not significantly influence results for bilingual participants. IRT posits that items serve as indicators of a latent variable, and individual differences on this latent trait influence observed item responses (e.g., Hambrick et al., 2010). A standard IRT analysis estimates both item parameters, such as difficulty and discrimination, and ability levels for each respondent.

IRT offers advantages over classical test theory (CTT) in several aspects (for reviews, see Embretson, 1996; Embretson & Reise, 2000). First, if its assumptions are met, an IRT model allows for scores on a latent ability continuum that are independent of the specific item pool (S. P. Reise & Waller, 2009). Second, IRT is one of several methods that enable the assessment of DIF, which occurs when different subgroups with the same latent ability have varying probabilities of responding to items (Stark et al., 2006). There are two types of DIF: uniform and nonuniform (Harpole, 2015). Uniform DIF affects the threshold parameter and indicates that the probability of endorsing an item is different across groups when matched at the latent trait level. Nonuniform DIF affects both threshold and discrimination parameters and indicates that the probability of endorsing an item is different across groups, but the exact relationship changes across the range of the latent trait continuum. The use of IRT and DIF analysis in vision science has been relatively rare but is gaining traction (e.g., Cho et al., 2015; Lee et al., 2015; Wilmer et al., 2012).

It is important to delineate the distinction between *DIF or item bias* on the one hand and *impact or latent mean differences* on the other. Impact refers to true differences in the latent trait distributions of the focal and reference groups, while DIF or item bias represents group differences caused by problems with the measurement instrument rather than true differences in ability (Stark et al., 2006). Because the accumulation of DIF across items can lead to biased overall test scores (i.e., differential test functioning (DTF), Chalmers et al., 2016; Stark et al., 2006), it impedes the ability to meaningfully compare different groups of examinees on a common scale (Smith & Reise, 1998). For example, in the presence of DIF, groups can conceivably differ in observed scores on a measure despite being equivalent on the latent ability. However, patterns of DIF across items are also possible that can produce (1) group equivalence on observed scores despite group differences on the latent ability, and (2) a cancellation effect such that DIF in one direction on some items is counterbalanced by DIF in the opposite direction in others. This pattern leads to significant DIF but no significant DTF (Lee et al., 2015; Stark et al., 2006). It is, of course, also possible for a test to exhibit no DIF or DTF (i.e., its psychometric properties are equivalent across groups) in the presence of true differences between groups in the latent ability.

The goal of Study 2 was to examine which of these alternatives was relevant to a comparison between native English and Spanish speakers in OR. Although recognizing that DIF could be found in some items, we predicted that, overall, DIF would be negligible. After adjusting the analyses for any items demonstrating DIF, we assessed whether the two groups differed in latent OR ability. Based on the results of Study 1 and the expectation that the language of participants should not influence object recognition ability, we predicted that the two groups would be generally equivalent. We should also note that in a typical DIF analysis, the groups are distinct but the test is identical, whereas in Experiment 2, both the groups and the language of instructions differ, providing two possible sources of DIF.

Our previous studies have indicated that three NOMTs tap into the same ability (Richler et al., 2017, 2019). In most applications, one or two of them would be sufficient, or they could be combined for more precise measurement. In Experiment 2, we used our resources to collect a larger sample of participants, using only two of the three categories. We selected those that produced measurements with the highest reliability and the more normal distributions in Experiment 1, Greebles, and Ziggerins.

### Method

#### Participants

Spanish- and English-speaking participants were recruited on Prolific.com in August 2021. Prolific had better options than Mechanical Turk to screen participants based on the language fluency they had declared on the general screener used on the platform. Participants were paid $2.75 for a median time of 18 min on the tasks ($9.17/hr). We recruited two groups of participants, with the only requirement that they be 18 years or older and their native language be English/Spanish. We did not know what the response rate would be for native Spanish speakers, so we did not constrain sampling in any other way. In total, we had 393 participants (267 women, 109 men, 17 not disclosed; mean age = 26.55 years, *SD* = 8.55, range = 18–57). The English-speaking group consisted of 193 (49.1%) participants with a mean age of 28.75 years (*SD* = 9.78). Their current country of residence was reported as UK (86), USA (75), Canada (17), other (9), and not reported (6). The Spanish-speaking group consisted of 200 (50.9%) participants with a mean age of 24.44 years (*SD* = 6.55). Their current country of residence was reported as Mexico (75), USA (58), Spain (29), Chile (23), other (7), and not reported (8). We had four attention checks in the study (where the two distractor objects were from very different categories in very different colors from the category currently tested) and discarded data from two additional participants, one from each group, for missing more than one attention check.

While the mean difference in age was only 4.3 years, the English-speaking group was older (*BF*_10_) = 26,353). There was, however, little evidence for a correlation between accuracy on the combined NOMTs and age in the sample (*r* = − 0.072, *BF*_10_ = 0.34). The proportion of women was larger in the English-speaking group (72%) than in the Spanish-speaking group (64%). There was moderate support for a difference in these proportions (*BF*_10_ = 2.98). However, we found moderate support for no difference between accuracy on the combined NOMTs and gender (*BF*_01_ = 4.48). We therefore ignored age and gender in the remainder of the analyses.

#### Measures

Participants performed two NOMTs (with Ziggerins and then Greebles) as in Study 1 and with all instructions in their native language. Given that Study 1 found evidence of strong and robust correlations between the NOMTs, especially the tests with novel categories called Greebles and Ziggerins (*r* = 0.772 and 0.741 for the English and Spanish instruction groups, respectively), we chose to combine the tests into a single measure to test for the presence of DIF and DTF.^1^ The NOMT uses a three-alternative forced-choice recognition task, making the probability of correctly answering a question by chance equal to 0.33. The NOMT consisted of 72 trials with Ziggerins and 72 trials with Greebles. Each item was scored as 0 (incorrect) or 1 (correct).

### Analysis and results

We present the analytical procedures and the results simultaneously to facilitate interpretation of the findings. All analyses described in the following sections were performed on the entire sample (*N* = 393). The sample was divided into two groups based on language proficiency (i.e., English-speaking and Spanish-speaking). Before we dive into IRT, we first consider a difference in performance between the groups that would be obvious in even the simplest analysis of the results. We discuss it now, before our more sophisticated analysis, because it allows us to raise some interesting challenges about motivation that can arise when comparing majority and minority populations on online platforms. Because the NOMT is not a speeded task, our item analyses consider only accuracy. But before we get to these item analyses, to further explore the question of differential motivation, we report analyses of response times to ensure that this measure did not hide larger differences between the two groups.

*Mean difference in accuracy between groups.* The Spanish-speaking group performed better (mean = 0.770, *SD* = 0.10) than the English-speaking group (mean = 0.722, *SD* = 0.10, BF_10_ = 3127, non-informative default Cauchy prior centered at 0 with an interquartile range of 0.707; all possible priors render *BF*_10_ > 18). This difference in mean performance between groups may appear surprising, and it was not theoretically expected, at least not on the basis of language of instructions or the participants’ linguistic identity (or any other identity variable that may vary between the samples, such as the small differences in age, gender, or country of origin). We believe the difference could be explained by the manner in which Prolific samples participants. On this platform, volunteers initially answer a questionnaire that surveys many dimensions, including primary language. Participants cannot see all the studies they are technically eligible for—instead, they only see the studies that the platform makes eligible to them specifically (Prolific clearly states that they use convenience sampling). The instructions to participants (Prolific.com, 2023) state that they attempt to “make study distribution fair so that participants get an equal chance to take part” and that for this reason, someone who has just participated in many studies may receive fewer offerings. Even with those efforts, individuals with a primary language other than English may be eligible for only a few studies most of the time. For instance, on October 2, 2023, with the sole filter of primary language being English versus Spanish, the site indicated that there were 27,961 eligible participants versus 653. Because participants on the platform are also often screened for the quality of their work, it is plausible that Spanish-speaking participants could be more motivated than English-speaking participants. Their performance on any of the relatively rare studies offered to them will have a higher impact (proportionally) on the availability of future studies.

This is an important consideration for any study that considers sampling online between groups that are differentially represented within a sampling system. It could have important limitations on any work attempting to compare performance in such groups. Critically, our goal here is not to ask whether these two groups of participants have the same average ability in object recognition. Rather, it is to ask whether all the known group differences in this study—the largest of which is the language of participants and test, but also any other differences (including small differences in age, gender proportion, country of origin, or motivation)—violate the measurement invariance of the tests. While we could have tried to control for some of these group differences in sampling (e.g., age and gender would be the easiest), group comparisons always come with the possibility of unexpected differences (e.g., differential effects of sampling on motivation are one example). As such, the specific situation we are considering here both exemplifies the challenges that can arise when combining data from different groups and sets a high bar for obtaining measurement invariance based on DIF analyses.

*Mean difference in response times between groups.* Before we proceeded with item-based analyses, we analyzed response times. Some work on the effect of motivation on measurement invariance has used fast response times as an indication for lack of effort (Rios, 2021). Research on the effect of motivation on measurement invariance often considers relatively large differences in motivation, such as those when the same time is given to students under high- versus low-stakes conditions (e.g., Wise & Kong, 2005). In contrast, the difference in motivation between groups in this study may be more limited, since all participants were paid and were screened with attention checks, and all of them knew that inattention could result in being penalized on the testing platform. The NOMT format uses accuracy and does not explicitly encourage speed, to avoid problems with the reliability of response times (Draheim et al., 2019). Nonetheless, differences in response time can reflect different strategies. Overall, performance on the test for all participants was positively related to median response time (*r* = 0.42, *p* < 0.001, *BF*_10_^2^ = 5.75E + 34, 95% CI = 0.33–0.50), suggesting a classic speed–accuracy trade-off. Despite this, the median response time (in ms) on the task was not different between groups, with moderate support for this conclusion (English *M*(*SD*) = 2693 (1147); Spanish *M*(*SD*) = 2817 (1043); *t* = 1.13, *p* = 0.26; *BF*_01_ = 4.85^3^). This gives us some confidence that we can evaluate measurement invariance of the NOMT using accuracy only (which is how the test has been used in the past).

*Item analyses.* To evaluate the psychometric properties of the NOMT and ultimately determine whether the NOMT exhibits measurement invariance across English- and Spanish-speaking participants, we performed a comprehensive analysis encompassing dimensionality, IRT, DIF, and DTF analyses.

*Dimensionality.* Before applying an IRT model and conducting DIF analyses, it is important to verify that the assumptions of unidimensionality and local independence are reasonably met. IRT models assume that the covariance among item responses can be explained by a single underlying latent trait, and item responses are uncorrelated after accounting for the latent trait. Although violations of unidimensionality and local independence can yield distorted item, person, and test parameter estimates, if the relationships among items are dominated by a single latent trait, then the impact of these minor dimensions may be small enough that multidimensionality can be safely ignored (D. Anderson et al., 2017). Therefore, researchers have developed methods to investigate whether data are “unidimensional enough” for IRT analyses.

First, we investigated the internal consistency of the NOMT by calculating omega hierarchical (${\upomega }_{\text{h}}$) and omega total (${\upomega }_{\text{t}}$) to determine the saturation of the items by a general factor (W. Revelle & Zinbarg, 2009; Schlegel & Scherer, 2016). ${\upomega }_{\text{h}}$ represents the proportion of variance due to a general factor that influences all of the items in a scale (e.g., the primary construct of interest). High values of ${\upomega }_{\text{h}}$ indicate that a significant portion of the item variance is explained by the general factor and suggest strong central construct saturation. ${\upomega }_{\text{t}}$ measures the proportion of the total variance that can be attributed to all factors in a model (the general factor plus any specific factors). ${\upomega }_{\text{h}}$ and ${\upomega }_{\text{t}}$ were calculated using the tetrachoric correlation matrix of the sample’s item responses in R version 4.0.2 (R Core Team, 2019) using the *psych* package (W. R. Revelle, 2024).

The results showed that 60% of the variance in the NOMT item responses is explained by one general factor (${\upomega }_{\text{h}}$), and 97% of the total variance in the NOMT item responses is explained by all factors (${\upomega }_{\text{t}}$). While additional specific factors explain more variance in the item responses, these results indicate that the saturation of the items by a general factor is strong.

The most commonly used method for assessing unidimensionality is exploratory factor analysis. A recommended guideline for determining whether a response matrix is sufficiently unidimensional is a ratio of the first to second eigenvalue of the correlation matrix that is greater than 3.0. A ratio greater than 3.0 provides evidence of a dominant general factor and indicates that, as a second step, bifactor exploratory factor analysis (EFA) should also be considered in the evaluation of dimensionality (Cho et al., 2015; Lee et al., 2015; S. Reise et al., 2011; S. P. Reise et al., 2010). A bifactor model is a latent structure that consists of one general factor and two or more orthogonal, specific factors. The general factor is conceptually broader and is generally the trait that the researcher is interested in scaling individuals on, while the specific factors tend to be more conceptually narrow and explain item response variance not accounted for by the general factor (Cho et al., 2015; S. P. Reise et al., 2010). The specific factors are often considered “nuisance” factors that interfere with the measurement of the construct of interest (S. P. Reise et al., 2010). If the factor loadings on the general factor of the bifactor model are similar to those from the unidimensional model, multidimensionality can be ignored and treated as nuisance (S. P. Reise et al., 2007). Additionally, Reckase (1979) found that when applying unidimensional IRT models to potentially multidimensional data, stable item parameter estimates can be obtained when the dominant factor accounts for at least 20% of the total variance.

Traditional EFA and the methods used to investigate dimensionality were originally developed for use with continuous data. The application of these techniques to categorical data (e.g., item factor analysis [IFA]) can lead to biased parameter estimates and fit statistics, making it more difficult to interpret results (Clark & Bowles, 2018). In the case of dichotomous data, the use of tetrachoric correlations and the mean- and variance-adjusted weighted least squares (WLSMV) estimator generally yields more accurate parameter and standard error estimates (Clark & Bowles, 2018; Garrido et al., 2016). However, results from simulation studies indicate that traditional model fit indices (e.g., RMSEA, CFI, TLI) and their commonly used cutoff values reported in the literature provide questionable utility in guiding decisions regarding dimensionality in the context of IFA (Clark & Bowles, 2018; Garrido et al., 2016). Given the difficulty associated with determining the appropriate dimensionality structure of dichotomous data, we took a multifaceted approach that included exploratory IFA and EFA on item parcels.

Prior to conducting these analyses, item response frequencies were inspected, and item 7 was removed from subsequent analyses because no participant in the Spanish-speaking group answered the item incorrectly. The first five eigenvalues of the sample’s tetrachoric correlation matrix were 29.19, 6.91, 6.06, 5.64, and 5.11, respectively. The first factor accounted for 20.41% of the total variance. The ratio of the first to the second eigenvalue was 4.22, providing evidence of a single strong common dimension. As such, bifactor IFA was used to explore the fit of models with more than one factor instead of regular IFA. Mplus version 8.00 (Muthén & Muthen, 2017) was used to fit the (bifactor) IFAs using tetrachoric correlations, the WLSMV estimator, and GEOMIN and BI-GEOMIN rotations. Model fit was evaluated using the following indices: root-mean-square error of approximation (RMSEA; Steiger & Lind, 1980), the comparative fit index (CFI; Bentler, 1990), and the Tucker–Lewis index (TLI; Tucker & Lewis, 1973). According to consensual criteria, RMSEA values less than 0.05 indicate good fit, and values less than 0.08 indicate acceptable fit (Hu & Bentler, 1999). CFI and TLI values greater than 0.95 indicate good fit, and values greater than 0.90 indicate acceptable fit (Hu & Bentler, 1999).

Based on the fit indices presented in Table 3, bifactor models consisting of seven or more factors provided an acceptable fit to the data across all three fit indices. RMSEA values indicated good fit across all models with relatively little variation across models. However, the results were difficult to interpret because of cross-loadings and factors defined by few items. The factor loadings in the general factor of the bifactor model with seven factors (average loading = 0.42) were only slightly different from the factor loadings of the unidimensional model (mean absolute deviation = 0.038), and the factor loadings of the general factor of the bifactor model were highly correlated with the factor loadings of the unidimensional models (*r* = 0.967). Furthermore, when interpretating the fit statistics of the IFA models, it is crucial to consider how certain characteristics of the data can exert differential effects of fit statistics and the effectiveness of their cutoffs (Clark & Bowles, 2018; Garrido et al., 2016). Although the RMSEA suggests an excellent fit for the unidimensional model, the corresponding CFI and TLI values fall below their typical recommended thresholds. However, the CFI and TLI may demonstrate poorer fit in models characterized by lower factor loadings, such as those around 0.40 (Clark & Bowles, 2018; Garrido et al., 2016). As the number of factors in an IFA solution increases, the difference between the reproduced data matrix and the observed data matrix will decrease. As a result, over-factored solutions (e.g., those with too many dimensions) will fit better than the correct solution according to commonly used model fit indices, such as the CFI and TLI (Clark & Bowles, 2018). Given that the average factor loading in the unidimensional model is 0.43, the relatively lower values of the CFI and TLI are to be expected.

**Table 3:** Exploratory item factor analysis results

Model	RMSEA [90% CI]	CFI	TLI
1-Factor	0.017 [0.015, 0.019]	0.773	0.770
2-Factor bifactor	0.014 [0.013, 0.017]	0.825	0.820
3-Factor bifactor	0.013 [0.011, 0.015]	0.855	0.848
4-Factor bifactor	0.012 [0.010, 0.015]	0.873	0.866
5-Factor bifactor	0.012 [0.008, 0.014]	0.891	0.883
6-Factor bifactor	0.011 [0.007, 0.014]	0.906	0.897
7-Factor bifactor	0.010 [0.007, 0.013]	0.916	0.907
8-Factor bifactor	0.010 [0.006, 0.013]	0.925	0.916

To provide an additional perspective on the question of a strong general factor, we used confirmatory factor analysis to examine the fit of a unidimensional model where parcels, rather than individual items, were used as indicators of a single latent variable (Sterba, 2011; Sterba & MacCallum, 2010). Item parceling involves the summing of several raw items to form a single, continuous score and is an effective strategy to investigate dimensionality when difficulties in evaluating the fit of latent variable models based on item-level data arise (Sterba, 2011). Items were randomly allocated 100 times to 12 parcels consisting of 11 or 12 items each. Specifically, the 71 Ziggerin items were randomly allocated into six parcels consisting of 11 or 12 items, and the 72 Greeble items were randomly allocated into six parcels consisting of 12 items. All 12 parcels loaded on a single latent trait. The CFI, TLI, and RMSEA of each model were calculated to determine the average fit statistic, as well as the standard deviation and range across the 100 models. The average RMSEA, CFI, and TLI were 0.038, 0.982, and 0.979, respectively, indicating good fit. The fit statistics across the 100 allocations were associated with low standard deviations and small ranges, indicating that the conclusions of acceptable model fit do not change across random allocations.

Taken together, these analyses indicate that the data are sufficiently unidimensional for IRT analyses and any observed multidimensionality due to item content or difficulty is ignorable.

### Item response theory analyses

Next, we compared the fit of a two-parameter logistic (2PL) unidimensional item response model with a three-parameter logistic (3PL) unidimensional item response model. The 2PL model predicts the probability of success for a person *s* on item *i* as

$$P\left({X}_{i}=\left.1\right|\theta \right)=\frac{\text{exp}\left[{a}_{i}\left(\theta -{b}_{i}\right)\right]}{1+\text{exp}\left[{a}_{i}\left(\theta -{b}_{i}\right)\right]},$$

where *θ* represents the trait level for person *s*, *b*_*i*_ is the difficulty of item *i*, and *a*_*i*_ is the discrimination for item *i* (Embretson & Reise, 2013). The 2PL model uses two parameters to describe item characteristics. Item discrimination parameters represent the ability of an item to differentiate between individuals with varying levels of ability. Larger discrimination parameter values indicate that the item is more sensitive to variations in the latent trait. Item location parameters represent the difficulty of an item. 3PL models incorporate an additional guessing parameter (*c*) that represents the probability of correctly responding to an item without any knowledge (i.e., by guessing; see Embretson & Reise, 2013). The 3PL model is appropriate for multiple-choice tests in which the probability of a correct response from an individual with very low ability (i.e., very low object recognition) may be significantly higher than zero because of random guessing or other factors such as distractors (De Mars, 2010; Diamond & Evans, 1973). The mathematical expression for the 3PL logistic function is

$$P\left({X}_{is}=\left.1\right|\theta \right)={c}_{i}+\left(1-{c}_{i}\right)\frac{\text{exp}\left[{a}_{i}\left(\theta -{b}_{i}\right)\right]}{1+\text{exp}\left[{a}_{i}\left(\theta -{b}_{i}\right)\right]}.$$

We examined whether a guessing parameter was needed to adequately describe the data, because the NOMT consists of three-alternative forced-choice items, making the probability of a correct response based on chance alone 0.333. However, estimates of the guessing parameter often differ from the random guessing probability (Embretson & Reise, 2013). Furthermore, 3PL models with unique guessing parameters for each item can have estimation problems, and it is often necessary to constrain the guessing parameter to be equal for all items or for groups of similar items (Embretson & Reise, 2013). While it is generally assumed that large sample sizes are needed for proper and accurate estimate of item parameters, particularly in the case of the 3PL model, simulation studies have found that fairly small samples can still be used without affecting the precision or accuracy of 3PL item parameters when tests are long (e.g., 30 or more items) (Akour & AL-Omari, 2013; Şahin & Anıl, 2017). One study found that sample sizes as small as 200 can yield acceptable 3PL item parameter estimates when combined with test length of 30 or greater (Akour & Al-Omari, 2013). Similarly, Sahin and Anil (2017) found that sample sizes of 350 resulted in accurate 3PL item parameter estimates when tests consisted of at least 30 items.

The 2PL and 3PL models were compared using the Akaike information criterion (AIC; Akaike, 1973), Bayesian information criterion (BIC; Schwarz, 1978), and the sample-size-adjusted Bayesian information criterion (SABIC; Sclove, 1987). Information criteria indices are based on some form of penalization of the likelihood function, with smaller values indicating better fit (Sen & Bradshaw, 2017). Note that the information indices differ in the specific penalty function applied to the likelihood. Specifically, the AIC penalizes the number of parameters in the model, while the penalty function for the BIC is based on the number of estimated parameters and the sample size. As a result, the BIC is more likely to select simpler models than the AIC. The SABIC is similar to the BIC but reduces the penalty placed on the sample size (Sen & Bradshaw, 2017).

All IRT and DIF analyses were conducted in R version 4.0.2 (R Core Team, 2019) using the *mirt* package (Chalmers, 2012). Specifically, the mirt() function was used to estimate the 2PL and 3PL models with full-information maximum likelihood estimation with an expectation–maximization (EM) algorithm (Bock & Aitkin, 1981; Bock & Zimowski, 1997; Chalmers, 2012). The mutipleGroup() function was used to compute anchored multiple-group IRT models, the DIF() function was used to determine whether DIF in an item was due to the discrimination or difficulty parameter, and latent trait scores were calculated using the fscores() function with expected a posteriori (EAP) estimation (Chalmers, 2012). Lastly, the DTF() function was used to examine differential test functioning (Chalmers, 2012).

Because, as anticipated, the 3PL model with unique guessing parameters for each item exhibited estimation problems, the guessing parameters were constrained to be equal across items (Embretson & Reise, 2013). The absolute fit indices for the 2PL model were RMSEA = 0.031, CFI = 0.888, and TLI = 0.887, while the restricted 3PL model yielded RMSEA = 0.032, CFI = 0.881, and TLI = 0.880. The 3PL model (AIC = 49,608.00, BIC = 50,748.49, SABIC = 49,837.84) with the guessing parameters constrained to be equal across items fit better than the 2PL model (AIC = 49,639.4, BIC = 50,775.91, SABIC = 49,868.44) according to all three information indices. We examined the item characteristics of the 3PL model with the guessing parameters constrained to be equal across items and found that items 128, 141, and 144 had negative item discrimination parameters, indicating that participants with lower ability had a higher probability of answering the items correctly than participants with higher ability. These items were removed from subsequent analyses. The item parameter estimates and standard errors of the 3PL model are presented in Table 4. Item discrimination estimates were variable, ranging from 0.097 to 3.592. According to guidelines proposed by Baker (2001), discrimination parameters are interpreted as follows: 0.01–0.34 (very low), 0.35–0.64 (low), 0.65–1.34 (moderate), 1.35–1.69 (high), and > 1.70 (very high). Based on these criteria, five items had very low discrimination parameters, 16 items had low discrimination parameters, 87 items had moderate discrimination parameters, 14 items had high discrimination parameters, and 18 items had very high item discrimination parameters. Item difficulty parameter estimates covered a wide range of ability levels, ranging from − 6.825 to 5.523. The item guessing parameter was estimated to be 0.240, which is lower than the expected random guessing probability of 0.333 in a three-alternative forced-choice task (Embretson & Reise, 2013). As a rule of thumb, standard errors of parameter estimates less than or equal to 0.75 are considered acceptable. The standard errors for the discrimination parameters were acceptable (range = 0.143 to 0.740) except for items 79 and 82. The majority of the standard errors for the threshold parameters were acceptable (range = 0.112 to 0.715), with the exception of the standard errors for items 11, 100, 137, and 143 (range = 3.743 to 8.818). Overall, these results indicate that the NOMT is satisfactory in its ability to discriminate between individuals of lower and higher ability levels across a broad range of the latent trait continuum. It is not surprising that some of the items exhibited suboptimal properties, and the item parameter estimates reported in Table 4 could be used to inform revisions of the NOMT (e.g., item selection and item removal). For example, items with low discrimination parameters and large standard errors are good candidates for item removal. Additionally, selecting items based on their discrimination parameters allows researchers to build tests that target specific ability ranges, facilitating the development of versions that are either more challenging or easier.

**Table 4:** Item parameter estimates (standard errors) of the three-parameter unidimensional item response model with item guessing parameters constrained to be equal across items

Item	a (*SE*)	b (*SE*)	c (*SE*)	Item	a (*SE*)	b (*SE*)	c (*SE*)
1	1.378 (0.342)	− 2.714 (0.486)	0.240 (0.027)	73	1.169 (0.346)	− 3.327 (0.758)	0.240 (0.027)
2	0.961 (0.298)	− 3.329 (0.855)	0.240 (0.027)	74	0.852 (0.236)	− 3.222 (0.762)	0.240 (0.027)
3	1.201 (0.324)	− 3.035 (0.623)	0.240 (0.027)	75	1.502 (0.336)	− 2.33 (0.368)	0.240 (0.027)
4	1.042 (0.459)	− 4.430 (1.574)	0.240 (0.027)	76	1.212 (0.34)	− 3.082 (0.657)	0.240 (0.027)
5	1.034 (0.401)	− 4.111 (1.284)	0.240 (0.027)	77	1.404 (0.396)	− 2.855 (0.576)	0.240 (0.027)
6	0.435 (0.156)	− 2.891 (1.034)	0.240 (0.027)	78	1.066 (0.259)	− 2.667 (0.524)	0.240 (0.027)
7				79	3.068 (1.240)	− 2.676 (0.404)	0.240 (0.027)
8	1.172 (0.415)	− 3.744 (1.018)	0.240 (0.027)	80	1.297 (0.332)	− 2.888 (0.544)	0.240 (0.027)
9	0.997 (0.314)	− 3.379 (0.873)	0.240 (0.027)	81	1.769 (0.446)	− 2.591 (0.409)	0.240 (0.027)
10	0.902 (0.338)	− 4.032 (1.272)	0.240 (0.027)	82	3.592 (1.372)	− 2.506 (0.305)	0.240 (0.027)
11	0.396 (0.246)	− 6.825 (4.077)	0.240 (0.027)	83	2.425 (0.702)	− 2.663 (0.370)	0.240 (0.027)
12	0.786 (0.357)	− 4.788 (1.902)	0.240 (0.027)	84	1.861 (0.478)	− 2.775 (0.422)	0.240 (0.027)
13	1.197 (0.392)	− 3.567 (0.888)	0.240 (0.027)	85	1.282 (0.701)	− 4.565 (1.870)	0.240 (0.027)
14	1.092 (0.330)	− 3.314 (0.792)	0.240 (0.027)	86	2.386 (0.653)	− 2.317 (0.321)	0.240 (0.027)
15	1.543 (0.476)	− 3.258 (0.67)	0.240 (0.027)	87	2.048 (0.592)	− 2.831 (0.451)	0.240 (0.027)
16	0.927 (0.276)	− 3.364 (0.835)	0.240 (0.027)	88	2.106 (0.740)	− 2.633 (0.512)	0.240 (0.027)
17	0.840 (0.290)	− 3.787 (1.124)	0.240 (0.027)	89	1.442 (0.376)	− 2.831 (0.515)	0.240 (0.027)
18	0.785 (0.285)	− 4.100 (1.296)	0.240 (0.027)	90	2.027 (0.661)	− 2.805 (0.512)	0.240 (0.027)
19	1.221 (0.237)	− 1.308 (0.239)	0.240 (0.027)	91	0.262 (0.143)	− 0.371 (0.609)	0.240 (0.027)
20	1.207 (0.275)	− 2.359 (0.423)	0.240 (0.027)	92	0.724 (0.186)	− 2.242 (0.542)	0.240 (0.027)
21	0.624 (0.180)	− 2.615 (0.715)	0.240 (0.027)	93	0.810 (0.18)	− 1.099 (0.289)	0.240 (0.027)
22	0.726 (0.177)	− 1.756 (0.429)	0.240 (0.027)	94	0.915 (0.184)	− 1.152 (0.259)	0.240 (0.027)
23	0.604 (0.167)	− 2.256 (0.612)	0.240 (0.027)	95	0.713 (0.175)	0.516 (0.249)	0.240 (0.027)
24	0.990 (0.219)	− 1.597 (0.332)	0.240 (0.027)	96	0.543 (0.177)	1.338 (0.463)	0.240 (0.027)
25	1.406 (0.290)	0.268 (0.144)	0.240 (0.027)	97	0.592 (0.172)	0.430 (0.284)	0.240 (0.027)
26	1.507 (0.288)	− 1.252 (0.208)	0.240 (0.027)	98	0.901 (0.193)	− 1.149 (0.277)	0.240 (0.027)
27	1.849 (0.334)	− 1.479 (0.192)	0.240 (0.027)	99	0.555 (0.170)	0.955 (0.372)	0.240 (0.027)
28	1.207 (0.224)	− 0.548 (0.171)	0.240 (0.027)	100	0.176 (0.207)	5.523 (6.348)	0.240 (0.027)
29	0.961 (0.200)	− 1.507 (0.304)	0.240 (0.027)	101	0.834 (0.186)	0.094 (0.203)	0.240 (0.027)
30	1.280 (0.233)	− 1.151 (0.206)	0.240 (0.027)	102	0.671 (0.160)	− 0.448 (0.256)	0.240 (0.027)
31	1.575 (0.285)	− 1.433 (0.208)	0.240 (0.027)	103	1.148 (0.214)	− 0.822 (0.194)	0.240 (0.027)
32	1.003 (0.198)	− 0.471 (0.189)	0.240 (0.027)	104	0.768 (0.189)	0.287 (0.220)	0.240 (0.027)
33	0.974 (0.195)	− 1.157 (0.252)	0.240 (0.027)	105	0.814 (0.205)	0.495 (0.220)	0.240 (0.027)
34	1.744 (0.331)	− 0.759 (0.157)	0.240 (0.027)	106	0.885 (0.225)	0.584 (0.211)	0.240 (0.027)
35	1.032 (0.204)	− 0.62 (0.199)	0.240 (0.027)	107	1.120 (0.235)	− 0.255 (0.175)	0.240 (0.027)
36	1.211 (0.243)	− 1.820 (0.305)	0.240 (0.027)	108	0.581 (0.198)	1.610 (0.516)	0.240 (0.027)
37	0.909 (0.225)	− 2.468 (0.529)	0.240 (0.027)	109	1.168 (0.218)	− 0.827 (0.194)	0.240 (0.027)
38	1.294 (0.238)	− 1.394 (0.228)	0.240 (0.027)	110	0.742 (0.187)	0.022 (0.225)	0.240 (0.027)
39	1.473 (0.257)	− 1.295 (0.196)	0.240 (0.027)	111	0.883 (0.301)	1.695 (0.400)	0.240 (0.027)
40	0.845 (0.201)	− 2.066 (0.452)	0.240 (0.027)	112	0.342 (0.196)	3.298 (1.775)	0.240 (0.027)
41	0.978 (0.192)	− 0.726 (0.208)	0.240 (0.027)	113	1.369 (0.228)	− 0.291 (0.141)	0.240 (0.027)
42	0.945 (0.213)	− 1.923 (0.391)	0.240 (0.027)	114	0.721 (0.172)	− 0.097 (0.229)	0.240 (0.027)
43	1.896 (0.362)	− 1.28 (0.184)	0.240 (0.027)	115	1.110 (0.218)	0.201 (0.165)	0.240 (0.027)
44	0.936 (0.234)	1.097 (0.257)	0.240 (0.027)	116	1.107 (0.221)	− 0.076 (0.167)	0.240 (0.027)
45	0.867 (0.210)	− 0.022 (0.202)	0.240 (0.027)	117	0.880 (0.189)	− 0.435 (0.212)	0.240 (0.027)
46	1.193 (0.259)	− 1.344 (0.273)	0.240 (0.027)	118	0.737 (0.175)	− 0.234 (0.232)	0.240 (0.027)
47	2.117 (0.504)	− 1.929 (0.274)	0.240 (0.027)	119	0.528 (0.256)	2.183 (0.847)	0.240 (0.027)
48	1.099 (0.203)	− 0.608 (0.183)	0.240 (0.027)	120	0.809 (0.192)	0.131 (0.209)	0.240 (0.027)
49	0.759 (0.174)	− 1.251 (0.326)	0.240 (0.027)	121	0.927 (0.204)	− 0.200 (0.195)	0.240 (0.027)
50	1.160 (0.207)	− 0.931 (0.195)	0.240 (0.027)	122	0.363 (0.196)	2.286 (1.150)	0.240 (0.027)
51	1.209 (0.245)	− 1.591 (0.28)	0.240 (0.027)	123	1.005 (0.219)	0.166 (0.177)	0.240 (0.027)
52	1.021 (0.230)	− 1.262 (0.291)	0.240 (0.027)	124	1.338 (0.275)	0.478 (0.153)	0.240 (0.027)
53	1.063 (0.214)	− 1.627 (0.298)	0.240 (0.027)	125	0.994 (0.226)	0.035 (0.182)	0.240 (0.027)
54	1.978 (0.323)	− 0.121 (0.112)	0.240 (0.027)	126	1.038 (0.201)	− 0.542 (0.190)	0.240 (0.027)
55	0.968 (0.197)	− 0.234 (0.185)	0.240 (0.027)	127	1.196 (0.242)	0.053 (0.160)	0.240 (0.027)
56	1.112 (0.251)	− 0.106 (0.173)	0.240 (0.027)	128			
57	2.518 (0.451)	− 0.543 (0.114)	0.240 (0.027)	129	0.426 (0.154)	0.378 (0.378)	0.240 (0.027)
58	2.015 (0.325)	− 0.750 (0.128)	0.240 (0.027)	130	0.596 (0.193)	0.654 (0.303)	0.240 (0.027)
59	1.557 (0.253)	− 0.742 (0.147)	0.240 (0.027)	131	0.321 (0.151)	1.715 (0.894)	0.240 (0.027)
60	1.175 (0.216)	− 1.221 (0.223)	0.240 (0.027)	132	0.975 (0.22)	0.569 (0.196)	0.240 (0.027)
61	0.386 (0.147)	− 0.663 (0.467)	0.240 (0.027)	133	0.640 (0.189)	1.264 (0.386)	0.240 (0.027)
62	1.169 (0.253)	− 1.300 (0.271)	0.240 (0.027)	134	0.745 (0.174)	0.079 (0.221)	0.240 (0.027)
63	1.642 (0.279)	0.040 (0.125)	0.240 (0.027)	135	0.753 (0.196)	0.208 (0.222)	0.240 (0.027)
64	1.892 (0.369)	− 1.212 (0.184)	0.240 (0.027)	136	0.749 (0.202)	0.976 (0.290)	0.240 (0.027)
65	1.008 (0.217)	0.696 (0.201)	0.240 (0.027)	137	0.385 (0.329)	4.964 (3.743)	0.240 (0.027)
66	1.362 (0.270)	− 1.942 (0.295)	0.240 (0.027)	138	0.358 (0.152)	0.300 (0.436)	0.240 (0.027)
67	0.892 (0.182)	− 1.418 (0.294)	0.240 (0.027)	139	0.867 (0.195)	0.010 (0.198)	0.240 (0.027)
68	1.334 (0.269)	− 0.556 (0.172)	0.240 (0.027)	140	0.767 (0.174)	0.189 (0.217)	0.240 (0.027)
69	1.715 (0.326)	− 0.493 (0.141)	0.240 (0.027)	141			
70	0.921 (0.185)	− 0.471 (0.201)	0.240 (0.027)	142	0.730 (0.183)	0.743 (0.266)	0.240 (0.027)
71	1.570 (0.418)	1.416 (0.217)	0.240 (0.027)	143	0.097 (0.153)	5.159 (8.183)	0.240 (0.027)
72	1.010 (0.210)	− 1.231 (0.266)	0.240 (0.027)	144			

Item parameter estimates (standard errors) of a three-parameter unidimensional item response model with 140 items. Results are based on 140 items after items 128, 141, and 144 were excluded because of negative item discrimination estimates in the initial analysis

#### Differential item functioning analyses

In DIF analyses, the group of interest is referred to as the focal group, while the group with whom the focal group is to be compared is termed the reference group. Here, the English- and Spanish-speaking groups were designated as the reference and focal groups, respectively. We used the IRT log-likelihood ratio (IRT-LR) all-others-as-anchors (AOAA) procedure paired with the NonsigMaxA criterion to examine whether items on the NOMT performed differently within the English- and Spanish-speaking samples (Lopez Rivas et al., 2009; Stark et al., 2006; Teresi et al., 2007; Thissen & Steinberg, 1988; Thissen et al., 1986; Wang & Woods, 2017). Like most IRT-based DIF detection methods, the IRT-LR test requires that estimates of item parameters obtained in different groups first be placed on a common metric before comparisons are made. This is typically done by specifying a set of anchor items that are assumed to be invariant across groups. However, when no prior information about DIF in the item set is available, the most popular anchor-selection strategy is the IRT-LR AOAA procedure. This procedure compares a baseline model in which all item parameters are constrained to equality across groups with a series of augmented models in which the parameters of the studied item are free to vary and all other items are constrained to equality across groups (e.g., the anchor item set). In this approach, the latent mean and variance of the reference group are fixed to zero and 1, respectively, while the latent mean and variance of the focal group are freely estimated. The difference between the log-likelihood statistics of the two models is distributed as a ${\chi }^{2}$ statistic with degrees of freedom equal to the difference in parameter estimates between the two models.

A notable limitation associated with the IRT-LR AOAA approach is that DIF items may be present in the anchor item set (Draheim et al., 2019; Meade & Wright, 2012; Wang & Woods, 2017). Contamination of the anchor item set (e.g., the presence of DIF items among the items used to link metrics) can interfere with DIF detection, leading to inflated type I/II errors and inaccurate parameter estimates (Cohen et al., 1996; Kim & Cohen, 1995; Lopez Rivas et al., 2009; Wang & Woods, 2017). The rate of type II errors increases as the magnitude of DIF and the number of DIF items among the anchors increases (Meade & Wright, 2012; Stark et al., 2006). The IRT-LR AOAA approach can be improved upon by pairing it with alternative anchor-selection procedures such as the NonsigMaxA criterion (Lopez & Rivas et al., 2009; Meade & Wright, 2012; Stark et al., 2006; Wang & Woods, 2017). The NonsigMaxA criterion is based on the finding that using highly discriminating items as anchors can yield higher power for DIF detection, especially in the case of small sample sizes (Lopez Rivas et al., 2009). For the NonsigMaxA criterion, after all items have been tested for DIF via the IRT-LR AOAA procedure, only the items with nonsignificant ${\chi }^{2}$ values are ranked based on their estimated reference group discrimination parameters in descending order, and then a certain number of items (typically less than 25% of the total number of items) with the largest discrimination parameters are selected as anchors (Wang & Woods, 2017). In the present study, our anchor item set consisted of 30 items with the highest discrimination parameters in the reference group with nonsignificant ${\chi }^{2}$ tests.

**Fig. 2:**
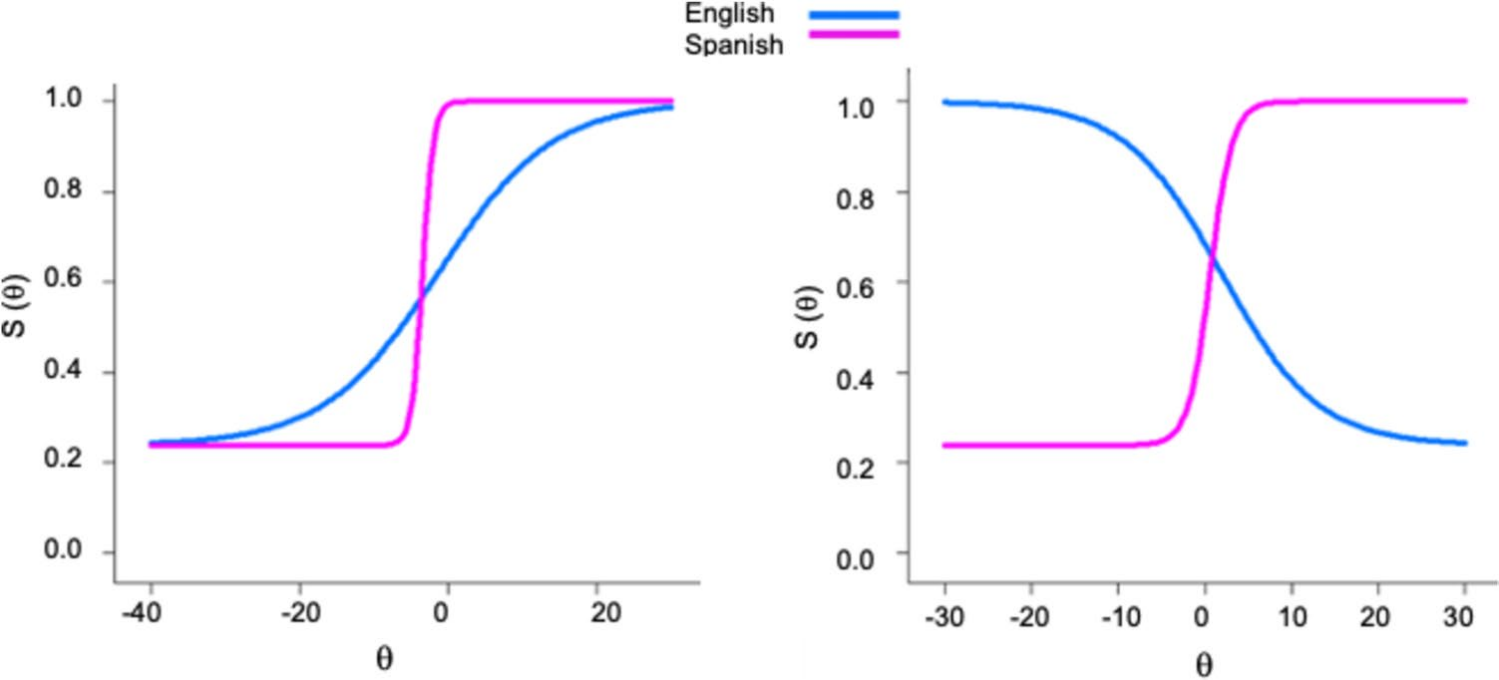
Item score functions of item 6 (left) and item 91 (right) by group. *Note.* DIF significant after B–H adjustment is bolded

Once the anchor item set was defined, all of the remaining (non-anchor) items were evaluated for DIF in the a- and b-parameters against this anchor set. If the omnibus test was significant, follow-up tests were conducted to determine whether the DIF was present in the a-parameters, b-parameters, or both. The Benjamini–Hochberg (B–H; Benjamini & Hochberg, 1995) method was used to adjust the critical *p*-value for multiple comparisons and reduce the possibility of type I errors (Edelen et al., 2006; Teresi et al., 2007). Using this approach, only two of the remaining 120 items were identified as having statistically significant DIF (item 6: ${\chi }^{2}$ = 88.614, *df* = 2, *p* < 0.001, B–H-adjusted *p* < 0.001; item 91: ${\chi }^{2}$ = 17.400, *df* = 2, *p* < 0.001, B–H-adjusted *p* = 0.012) (see Table 5). The parameter-specific tests indicated that item 6 exhibited significant DIF in the threshold parameter (uniform DIF), while item 91 exhibited significant DIF in both the discrimination and threshold parameters (nonuniform DIF). Item score functions of the DIF items are presented in Fig. 2.

**Table 5:** Results of DIF analyses between English- and Spanish-speaking groups

Item	Group	$\alpha$(*SE*)	$\beta$(*SE*)	$\gamma$(*SE*)	Overall DIF ${\upchi }^{2}$ (*df* = 2)	*p*-value	$\alpha$ DIF ${\upchi }^{2}$ (*df* = 1)	*p*-value	$\beta$ DIF ${\upchi }^{2}$ (*df* = 1)	*p*-value
6	English	0.130 (0.185)	− 1.356 (2.441)	0.239 (0.028)	88.614	** < .001**	3.039	.081	81.172	** < .001**
	Spanish	1.350 (0.695)	− 3.443 (1.418)	0.239 (0.028)						
91	English	− 0.180 (0.196)	1.909 (2.345)	0.239 (0.028)	17.400	** < .001**	12.694	** < .001**	8.065	**.005**
	Spanish	0.784 (0.237)	0.652 (0.286)	0.239 (0.028)

Next, multigroup IRT analyses were conducted to obtain separate item and subject information (Lee et al., 2015). In multigroup IRT models, item parameters of each group are estimated simultaneously, and the item parameters estimates of the groups are connected to a common (latent) metric using non-DIF items (Lee et al., 2015). Specifically, we fit a multiple-group 3PL model in which item parameters of the DIF-free items were constrained to equality across groups, and items that exhibited DIF had group-specific item parameters. In the presence of anchor items, the multigroup IRT model can be identified by specifying a standard normal distribution (*M* = 0, *SD* = 1) for the latent variable of the reference group (i.e., the English-speaking group). This also allows for the mean and variance of the latent variable of the focal group (i.e., Spanish-speaking group) to be estimated. The results indicated that the Spanish-speaking group had a higher latent mean (*M* = 0.488, *p* < 0.001) than the English-speaking group. The variances of the latent trait in the English- and Spanish-speaking groups were not significantly different (*S*^2^ = 1.112, *p* = 0.465).

#### Differential test functioning analysis

While individual items may exhibit statistically significant DIF, the cumulative effect of DIF on the total scores across groups may be negligible. To determine the magnitude of the DIF effects on the aggregated score of the NOMT, we conducted differential test functioning (DTF) analyses and examined the expected test score functions and test information functions (Chalmers et al., 2016). The signed DTF (*sDTF*) statistic measures systematic test scoring bias and indicates whether one group is consistently scored higher across a specified range of the latent variable (Chalmers et al., 2016). The magnitude of test scoring bias can range from − TS to + TS, where TS represents the highest possible test score, which is 140 for the present version of the NOMT. The unsigned DTF (*uDTF*) statistic represents the average area between the two tests curves and indicates the degree to which absolute deviations in item parameter estimates are aggregated across the entire test (Chalmers et al., 2016). The *uDTF* statistic ranges from 0 to TS. Statistical significance of either of these DTF statistics indicates that nontrivial DTF is present due to non-invariant items identified in the DIF analyses. The impact of DIF items was also examined by comparing IRT scale scores and raw total scores with and without items identified as having DIF.

**Fig. 3:**
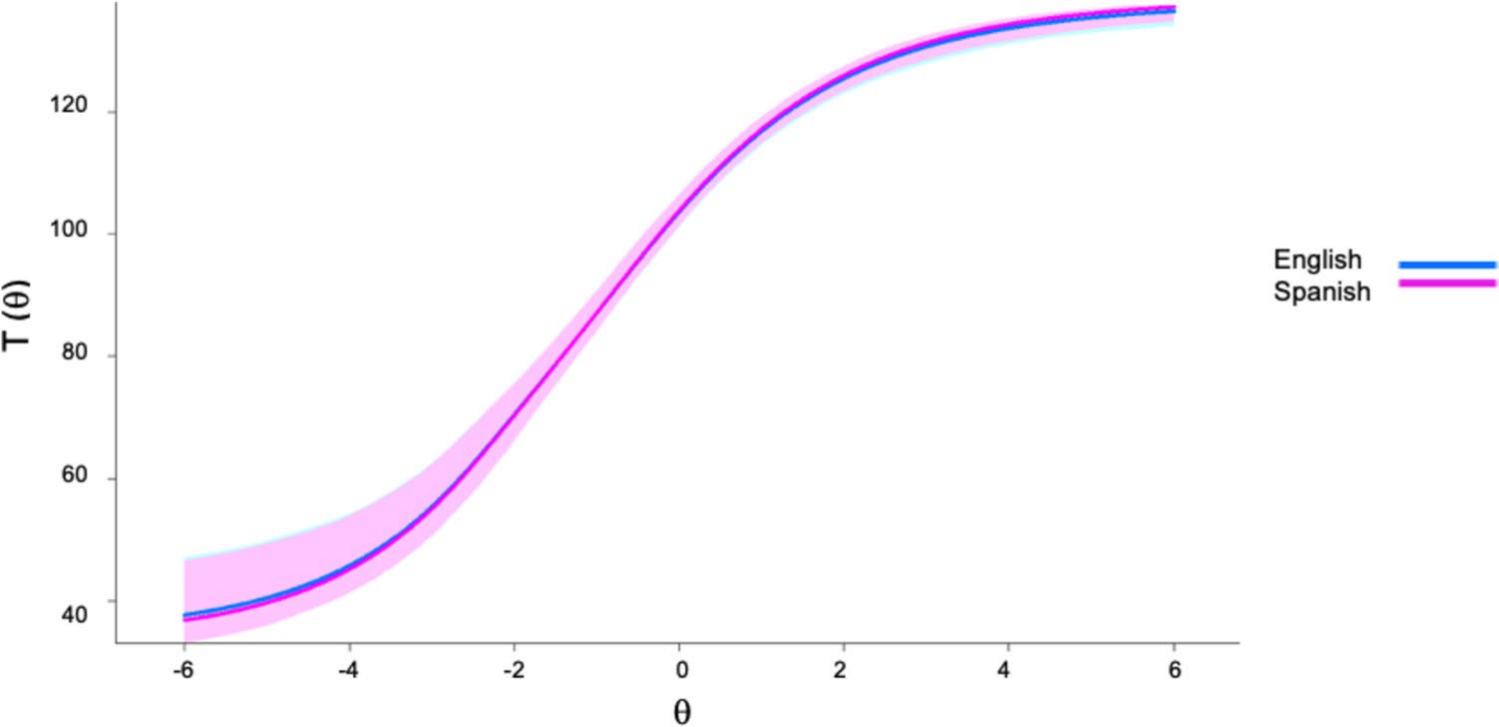
Expected NOMT scores as a function of latent trait levels of English- and Spanish-speaking participants with 95% confidence intervals

Figure 3 shows that there is substantial overlap in the confidence region for the two groups’ expected total scores across the range of the latent trait continuum, meaning that when English- and Spanish-speaking individuals are matched on the underlying latent trait, they receive nearly equivalent raw total scores on the NOMT. The signed and unsigned DTF analyses help quantify the overall degree of bias in the total scores. The respective results indicated no significant test bias (*sDTF* = − 0.085, 95% CI [− 0.277, 0.054], *p* = 0.324; *uDTF* = 0.469, 95% CI [0.209, 0.722]). Specifically, the *sDTF* statistic represents a bias in the total scores of approximately 0.085 raw points in favor of the Spanish-speaking group.

**Fig. 4:**
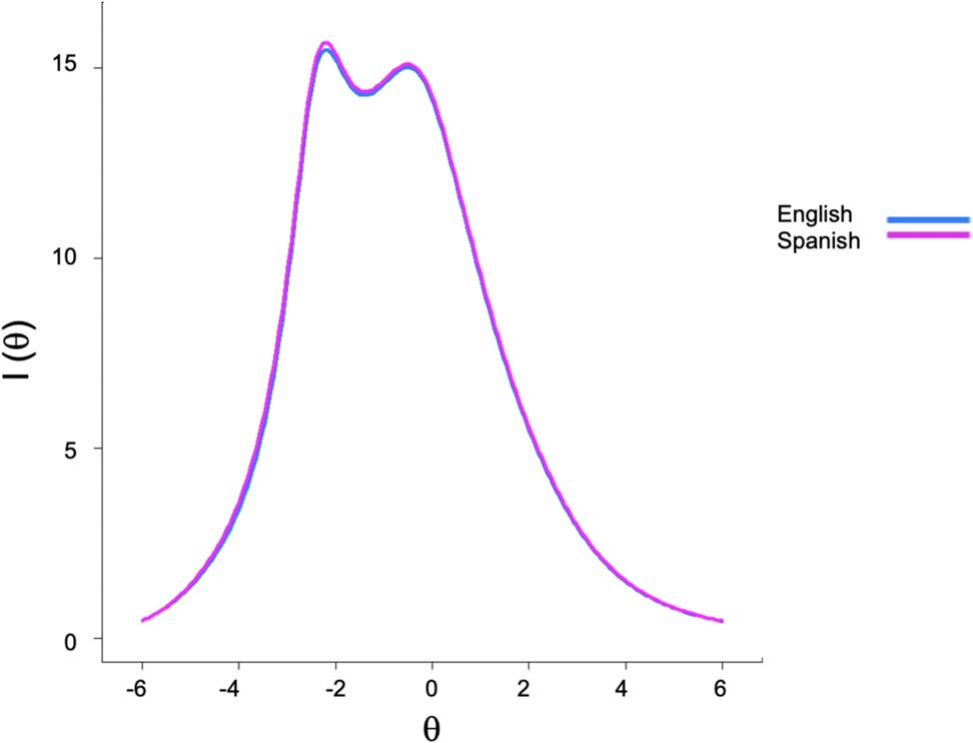
Test information as a function of latent trait levels of English- and Spanish-speaking participants

As indicated in Fig. 4, and consistent with DTF being nonsignificant, the test information functions for the two groups are almost identical. Test information functions are derived from item parameter estimates and indicate how precise a scale is across the latent trait continuum. The identical test information functions indicate that the NOMT is equally precise in measuring the latent trait in both groups. We computed the overlap coefficient (OC; e.g., Anderson et al., 2012; Schmid & Schmidt, 2006) indicating the degree of similarity between the two curves. To make the OC values more interpretable, we first rescaled the test information values of each group so that the total area under each curve equaled 1. Using numerical integration via Simpson's rule, we then computed the OC as the area under the curve subtended by the minimum function value across the two groups at each point in the ability continuum. Computed in this manner, the OC can take on values between 0 and 1, with 1 indicating identical function values that completely overlap. The observed OC = 0.996 indicates that the test information curves for the two groups were, for all practical purposes, identical. This is not surprising given that the great majority of the item parameters are constrained to be equal across the two groups in the final model that allowed for DIF on a small minority of items (e.g., items 6 and 9).

The influence of the DIF items was also investigated by comparing IRT latent trait scores and standardized raw total scores with and without items detected as having DIF for each of the groups. Multigroup 3PL item response models were used to estimate latent means in the English- and Spanish-speaking groups. Several variations of latent trait scores were calculated: (1) IRT scale scores (ignoring DIF) are based on a 3PL multigroup model where the item parameters are constrained to equality across groups, the latent mean and variance of the English-speaking group are set to 0 and 1, respectively, and the latent mean and variance of the Spanish-speaking group are freely estimated; (2) IRT scale score (language calibration) are based on a 3PL multigroup model where DIF items are allowed to vary across groups and all other item parameters are constrained to equality, the latent mean and variance of the English-speaking group are set to 0 and 1, respectively, and the latent mean and variance of the Spanish-speaking group are freely estimated; (3) IRT scale scores (DIF items removed) are based on a 3PL model using only non-DIF items where item parameters are constrained to equality across groups, the latent mean and variance of the English-speaking group are set of 0 and 1, respectively, and the latent mean and variance of the Spanish-speaking group are freely estimated. Raw total scores of the NOMT were standardized to facilitate the comparison between scores based on the full set of items and scores based on only non-DIF items.

Table 6 shows that the mean scores of the Spanish-speaking group are consistently higher than the mean scores of the English-speaking group and that the effect sizes of the mean differences are not affected by the DIF items (Cho et al., 2015; Smith & Reise, 1998). We also found that the IRT scale scores using a calibration by group (e.g., IRT scale scores language calibration) and scores using a calibration with all subjects (e.g., IRT scale scores ignoring DIF) were highly correlated (*r* = 0.999, *p* < 0.001), indicating that the relative ordering of individuals’ scores does not change when using a separate scaling for English- and Spanish-speaking individuals (Cho et al., 2015; Smith & Reise, 1998). Therefore, DIF items do not significantly distort the scale scores at the group level, and English- and Spanish-speaking individuals can be scored and compared on the same scale in the presence of DIF items.

**Table 6:** Group mean differences with and without DIF items

Scores	English-speaking *M* (*SD*)	Spanish-speaking *M* (*SD*)	$t(391)$	*p*	Effect size [95% CI]
IRT scale scores (ignoring dif)	0.00 (0.96)	0.50 (1.02)	5.03	< .001	0.51 [0.31, 0.71]
IRT scale scores (language calibration)	0.00 (0.96)	0.49 (1.01)	4.91	< .001	0.50 [0.29, 0.70]
IRT scale scores (DIF items removed)	0.00 (0.96)	0.49 (1.01)	4.90	< .001	0.50 [0.29, 0.70]
Standardized total scores (all items)	− 0.23 (0.98)	0.22 (0.97)	4.65	< .001	0.47 [0.27, 0.68]
Standardized total scores (DIF items removed)	− 0.23 (0.99)	0.23 (0.96)	4.51	< .001	0.46 [0.25, 0.66]

## Discussion

In Study 2, we investigated whether NOMT items (for Ziggerins and Greebles) and the test with both sets of items as a whole function equivalently across English- and Spanish-speaking groups, using IRT DIF analysis. Before applying an IRT model, we first examined the dimensionality of the NOMT and found that a unidimensional model adequately explained the item response variance. Next, we compared several IRT models and found that the 3PL model with equal guessing parameters provided the best fit to the data. Only two items were identified as having significant DIF (out of 140), and they did not distort scale scores at the group level. Together, these results suggest that the NOMT functions equivalently for English- and Spanish-speaking groups and can produce meaningful group comparisons. We obtained evidence of equivalent functioning of the NOMT in these groups in the face of an overall difference (in both raw and latent scores) that we speculated may be due to motivational differences caused by differential access to online studies. This explanation would have to be evaluated further, as it could have general implications whenever a minority and a majority group are compared on online platforms. Evidence that the language difference does not preclude comparison of a single continuum of individual differences in object recognition on the NOMT provides a stronger footing for exploring other issues that can influence performance on the test.

## General discussion

In Study 1, we found that English–Spanish bilinguals, when randomly assigned to the English or Spanish version of novel object memory tests (NOMTs) for three different categories of novel objects, exhibit similar overall performance. The advantage of random assignment is that, had we found any difference between the two versions, they could not have been attributed to participants instead of instructions.

However, in practice, test versions in different languages are particularly important when participants are not bilingual. A relatively new idea for vision science, but one that is growing in other fields (Jones et al., 2020; Karr et al., 2023; Langa et al., 2020), is that of combining information from different studies across different populations to increase sample sizes and generalizability. While tests in vision science are mostly image-based, they nonetheless require some instructions, and in the context of the growing trend of online testing, there is often little opportunity to ensure that participants have understood the instructions. When psychologists apply a test across different groups or populations or use the same test online and in the lab, they want assurance that the test can measure the same ability across these groups or situations. For instance, Cho et al. (2015) used DIF analyses to assess whether a test of face recognition ability (the Cambridge face memory test, or CFMT) measured the same ability across gender differences, age groups, and in online versus laboratory testing conditions. Our approach in Study 2 is similar but differs in that both test versions and populations differ at the same time, such that any difference between groups could be attributed to either the language of instructions or the participants (e.g., their ability, their motivation, other variables we did not measure). If we only had Study 2, a possible explanation for the group difference could have been that our instructions in the two languages were not equivalent (one could have been more confusing, or less motivating, than the other). Randomly assigning the same bilingual participants to both sets of instructions in Study 1 rules this out. However, because we built on the results of Study 1, we can assume that participants are the main possible source of DIF between our groups. Our results provided good evidence that people recruited online as English or Spanish speakers tested in the NOMT version that matches their primary language can be meaningfully compared. Note that this is in spite of finding a group difference on the NOMT which we provisionally attribute to differential motivation due to a factor other than the instructions.

The presence of a significant mean difference between the two groups in the absence of DIF also suggests that the difference cannot be attributed to the test. It either represents a true difference in ability or the role of some other external factor. Here, we were more interested in DIF than the group difference, but future work could investigate the explanation for this difference (and should devote more effort to matching the groups on age, gender, and country of origin when doing so). While a difference of language of origin on visual ability is not to be expected theoretically, our results fortuitously highlighted a very important factor when comparing groups sampled using Prolific (or similar platforms). Motivation is a possible influence on virtually any performance test, including intelligence tests, where it can affect predictive validity (Duckworth et al., 2011). Studies that use the NOMT to measure object recognition ability can use performance on other performance tests (for instance IQ tests) to attempt to control for differences in motivation (Chow et al., 2021, 2023).

While our study focused specifically on language-based DIF, future work should examine other participant variables that could affect measurement invariance, including age or gender. Research on object recognition measures has so far shown these factors to be more important for familiar categories than for novel objects. For instance, a study comparing three tasks for three novel object categories and three familiar object categories (birds, planes, and transformer robots) found the only evidence for a gender effect was in one of the nine tasks, for transformer robots (Sunday et al., 2021). The role of participant characteristics not only appears to be stronger for familiar than novel objects, but also appears to vary among familiar categories. For instance, age effects (in the form of DIF) were also found to be stronger for cars than for faces (Cho et al., 2015; Lee et al., 2015), although this may not be true of all test formats (Sunday et al., 2018a, 2018b). Because participant characteristics have so far been found to influence object recognition performance only for familiar categories, a reasonable hypothesis is that such effects are mediated by interest and experience. Testing these effects across demographic groups could strengthen support for visual tests’ unbiased use, regardless of participant background.

A limitation of the present approach is that DIF analyses require large samples and specialized analyses that not all experimenters are familiar with. We would argue that, early in the process of adopting a new measurement approach, a careful analysis like the one we present here is particularly important. A comparison of the sum scores or their distributions across different populations would not be sufficient to ensure that the same ability is tested across test versions. But work like ours can serve as a model for similar analyses in other tasks and as a proof of concept that equivalence is possible (and perhaps, given additional studies on other tasks, is to be expected). For example, concerns about equivalence under online testing conditions were more important when this approach was novel. Many studies (e.g., Cho et al., 2015; Crump et al., 2013; Germine et al., 2012) are devoted to evaluating the validity of online versions of tests traditionally performed in the laboratory, but experts are now much less likely to question the approach, even if a specific test has never been systematically compared in the two settings. We believe that in the context of the measurement of visual abilities where instructions are a relatively minor, albeit necessary, part of the test, this could be the future. Tests could be more routinely translated and samples tested with instructions in different languages could be combined to support one of the critical goals of psychology in the twenty-first century, which is to reduce inequities and foster diversity in our science and its applications.

## Data Availability

The materials and datasets generated during and analyzed during the current study are available in the figshare repository: 10.6084/m9.figshare.24395098.v2.

## Footnotes

1 IRT models are relatively robust (i.e., item parameters are well recovered and latent trait estimates reflect individual differences on the target latent trait dimension) to violations of unidimensionality if the multidimensionality is due to multiple latent dimensions that are moderately correlated (*r* > .40) (Reise et al., 2014; Kirisci et al., 2001).

2 This is using a non-informative stretched beta prior of 1, although the result holds for priors of .003 to 2.

3 This is using a non-informative Cauchy prior of .707, and BF_01_ was > 3 for all priors larger than .4.

## Code availability

N/A.

## Declarations

### Open practices statement

This study was not preregistered. The materials and datasets generated during and analyzed during the current study are available in the figshare repository: 10.6084/m9.figshare.24395098.v2.
